# Interaction of sleep quality and psychosocial stress on obesity in African Americans: the Cardiovascular Health Epidemiology Study (CHES)

**DOI:** 10.1186/1471-2458-10-581

**Published:** 2010-09-28

**Authors:** Aurelian Bidulescu, Rebecca Din-Dzietham, Dorothy L Coverson, Zhimin Chen, Yuan-Xiang Meng, Sarah G Buxbaum, Gary H Gibbons, Verna L Welch

**Affiliations:** 1 Morehouse School of Medicine, Atlanta, GA, USA; 2 Jackson State University, Jackson, MS, USA

## Abstract

**Background:**

Compared with whites, sleep disturbance and sleep deprivation appear more prevalent in African Americans (AA). Long-term sleep deprivation may increase the risk of obesity through multiple metabolic and endocrine alterations. Previous studies have reported contradictory results on the association between habitual sleep duration and obesity. Accordingly, we aimed to assess whether sleep quality and duration are inversely associated with body mass index (BMI) and obesity and test whether these associations are modified by psychosocial stress, known to influence sleep quality.

**Methods:**

A sample of 1,515 AA residents of metropolitan Atlanta, aged 30-65 years, was recruited by a random-digit-dialing method in 2007-08. The outcome obesity was defined by BMI (kg/m^2^) continuously and categorically (BMI ≥ 30 versus BMI < 30). Global sleep quality (GSQ) score was computed as the sum of response values for the seven components of the Pittsburgh Sleep Quality Index (PSQI) scale. GSQ score was defined as a continuous variable (range 0-21) and as tertiles. The general perceived stress (GPS), derived from the validated Cohen scale, was categorized into tertiles to test the interaction. Chi-square tests, correlation coefficients and weighted multiple linear and logistic regression were used to assess the associations of GSQ, GPS and obesity.

**Results:**

The mean (standard deviation) age was 47.5 (17.0) years, and 1,096 (72%) were women. GSQ score categorized into tertiles was associated with BMI. Among women, after multivariable adjustment that included age, gender, physical activity, smoking status, education, total family income, financial stress and history of hypertension, hypercholesterolemia, diabetes and myocardial infarction, obesity was associated with sleep quality as assessed by GSQ continuous score, [odds ratio, OR (95% C.I.): 1.08 (1.03 - 1.12)], and with a worse sleep disturbance subcomponent score [OR (95% C.I.): 1.48 (1.16 - 1.89)]. Among all participants, stress modified the association between obesity and sleep quality; there was an increased likelihood of obesity in the medium stress category, OR (95% C.I.): 1.09 (1.02 - 1.17).

**Conclusion:**

Sleep quality was associated with obesity in women. The association of sleep quality with obesity was modified by perceived stress. Our results indicate the need for simultaneous assessment of sleep and stress.

## Background

Long-term sleep deprivation may increase the risk of obesity through multiple metabolic and endocrine alterations [1-11]. Previous studies have showed that, compared with whites, African Americans (AA) have greater sleep deprivation as well as more sleep disturbance [12-16]. Along with the resulting increase in obesity, they threaten to widen the racial disparity in cardiovascular disease (CVD). Studies such as Whitehall II[17] and Sleep Heart Health Study[18] found no association between habitual sleep duration and obesity, whereas other studies such as NHANES I[19], the Zurich cohort[20], CARDIA[16] and the Nurses Health Study[21] suggested the contrary. Generalizability of these findings is questionable because those cohorts' participants, with the exception of NHANES, had normal or high-normal weight.

Known to have an increased prevalence in AA[22], psychosocial stress has also been associated with CVD[23] and obesity[24] in both whites and AA [25,26]. Interestingly, the literature on stress and sleep deprivation indicates that there might be a bidirectional relationship between these two variables ([27,28]). With the high prevalence of long-term sleep deprivation, the known adverse effects of psychosocial stress on cardiovascular disease may have a more detrimental effect if proven to interact with sleep deprivation [29,30]. Consequently our aim was to test whether habitual sleep is inversely correlated with body mass index (BMI) and obesity. We also aimed to test whether the above putative association is modified by psychosocial stress, i.e., the association between sleep and BMI varies by levels of stress. We also tested the individual sleep components' association with obesity separately by gender because gender is known to be associated with sleep disturbance [31-33].

## Methods

The Cardiovascular Health Epidemiology Study (CHES) includes a random sample of 1,515 AA residents in four counties of the metropolitan Atlanta area. The participants were aged 30-65 years and recruited in 2007-2008 through a random-digit-dialing (RDD) method for telephone survey of health behaviors. The RDD was conducted by the Southern Research Group, a contractor experienced with national telephone surveys including surveys sponsored by the Centers for Disease Control. Neighborhood median income was used as a stratifying variable to more efficiently evaluate the within-ethnic group effect of neighborhood characteristics, yielding 8 sampling frames. U.S. Census information[34] was thus used to obtain our weighted study sample.

The outcome variable obesity was defined by BMI (kg/m^2^), categorically (BMI ≥ 30, defining obesity; BMI between 25 and 30, corresponding to overweight, and BMI < 25, defining normal weight) and continuously, using the self-reported height and weight. Global sleep quality (GSQ) score, the exposure variable, was computed as the sum of response values for the seven components of the Pittsburgh Sleep Quality Index (PSQI) scale[35] (sleep quality, sleep latency, sleep duration, habitual sleep efficiency, sleep disturbance, use of sleeping medication and daytime dysfunction). The GSQ score was defined as a continuous variable (range 0-21, with higher scores reflective of poorer sleep quality) and as a trichotomous variable using the tertiles (obtained using ranking with ties). The individual PSQI components were also evaluated. Sleep duration was assessed using the third component of the Pittsburgh questionnaire that queried how many hours of actual sleep the participants got at night during the previous month. We composed two variables, one that used the actual number of hours (sleep duration as a continuous variable) and one that used the sleep duration categories defined by the Pittsburgh Sleep Quality component: more than 7 hours, between 6 and 7 hours, between 5 and 6 hours and less than 5 hours. The general perceived stress (GPS) was composed using the 14 questions from the validated Cohen scale (with a range of 0 to 64 points, with higher scores indicating higher stress)[36], and categorized into tertiles to test the interaction. Total family income was categorized into four categories: less than $25,000, between $25,000 and $49,999, between $50,000 and $74,999 and $75,000 or more. Financial stress was composed using a scale[37,38] composed of 5 questions (with a range of 0 to 12 points, with higher scores indicating higher stress), scale that was validated previously in surveys such as NHANES. Additional self-reported covariates that were included in the study included age, gender, physical activity (dichotomized, yes or no), smoking (current, former or non-smoker), education (categorized in three categories, less, equal and more than a high school education) and CVD comorbidity (self-reported history of hypertension, diabetes, hypercholesterolemia or myocardial infarction). Physical activity was queried (yes or no) with the question: "During the past month, other than your regular job, did you participate in any physical activities or exercises such as running, calisthenics, golf, gardening, or walking for exercise?"

The study was approved by the Morehouse School of Medicine's Institutional Review Board. Verbal informed consent was also obtained from the study participants by SRG before initiating the survey.

To assess the association between the variables considered in our analyses, Spearman correlation coefficients were used to account for the categorical nature of the PSQI components. Chi-square tests were used to assess the statistical significance between the categories. Tests for interaction between gender and global sleep quality general score and PSQI subcomponents were used to examine whether the association of obesity with each correlate differed between men and women. Weighted multiple linear and logistic regression models were also used to assess the independent association of GSQ score and obesity, adjusting for covariates and calculating correct variances given the complex weighted structure of the study sample. The association between the actual number of hours of sleep and obesity was also assessed. Statistical significance was set at p < 0.05 for the main and the interactive effects. SAS statistical software version 9.2 (with its survey procedures) was used for the analysis (SAS Institute Inc., Cary, NC, USA).

## Results

In our sample of exclusively AA, the mean (standard deviation; SD) age was 47.5 (17.0). For BMI, the mean (SD) was 29.4 (7.1). 557 participants (36.6%) were younger than 50 years of age and 1,096 (72%) were women. Fifty percents of those surveyed reported sub-optimal sleep (duration < 6 hours/night). Over 50% of respondents had poor quality sleep (GSQ score > 5).

The main characteristics of study participants by tertiles of GSQ, by tertiles of GPS and by sleep duration are presented in Table 1; with few exceptions such as age, hypertension and diabetes, there was a significant association between those characteristics (such as gender, physical activity, education, history of myocardial infarction, financial stress and family income) and sleep quality. Obesity was highly significantly associated with GSQ (p < 0.0001). There was an association between sleep duration and age, categorized BMI (p = 0.03), smoking status, physical activity and history of diabetes and myocardial infarction (Table 1). Sleep duration was also highly associated with financial stress and total family income (Table 1). GPS was associated with age, smoking status, physical activity, education, history of myocardial infarction, financial stress and family income (Table 1).

**Table 1:** Characteristics of study participants according to global sleep quality, general perceived stress and sleep duration*

	Global Sleep Quality Score			**P for trend**^‡^
	**High Quality Sleep** **(lowest tertile)**	**Medium Quality Sleep** **(medium tertile)**	**Low Quality Sleep** **(upper tertile)**	

Score Range	0 - 4	5 - 7	8 - 19	

N	576	403	536	

Age				0.13
< 50 years	206 (35.8)	170 (42.2)	205 (38.3)	
≥ 50 years	370 (64.2)	233 (57.8)	331 (61.7)	
Gender				<0.0001
Men	182 (31.6)	133 (33.0)	112 (20.9)	
Women	394 (68.4)	270 (67.0)	424 (79.1)	
BMI, kg/m^2^				<0.0001
≤ 25	162 (29.4)	90 (23.2)	122 (23.6)	
> 25 and < 30	215 (39.0)	146 (37.6)	152 (29.3)	
≥ 30	174 (31.6)	152 (39.2)	244 (47.1)	
Smoking Status				0.05
Current Smoker	76 (13.3)	53 (13.2)	101 (18.9)	
Former Smoker	118 (20.7)	89 (22.1)	97 (18.1)	
Non-smoker	377 (66.0)	260 (64.7)	337 (63.0)	
Physical Activity^§^				<0.0001
Yes	461 (80.4)	286 (71.0)	374 (70.0)	
No	112 (19.6)	117 (29.0)	160 (30.0)	
Education^#^				0.02
Less than HS	53 (9.2)	28 (7.0)	68 (12.8)	
HS graduate	132 (23.0)	101 (25.0)	138 (25.9)	
More than HS	390 (67.8)	274 (70.0)	326 (61.3)	
Hypertension^†^				0.17
Yes	314 (54.5)	225 (56.1)	320 (60.0)	
No	262 (45.5)	176 (43.9)	213 (40.0)	
Diabetes^†^				0.02
Yes	92 (16.0)	54 (13.5)	108 (20.3)	
No	484 (84.0)	345 (86.5)	425 (79.7)	
High Cholesterol^†^				0.02
Yes	200 (34.8)	154 (38.6)	226 (43.1)	
No	375 (65.2)	245 (61.4)	299 (56.9)	
History of MI^†^				0.0003
Yes	30 (5.2)	14 (3.5)	51 (9.6)	
No	545 (94.8)	388 (96.5)	482 (90.4)	
Financial Stress				<0.0001
Lowest tertile	272 (47.2)	132 (32.8)	126 (23.5)	
Middle tertile	186 (32.3)	131 (32.6)	155 (29.9)	
Upper tertile	118 (20.5)	139 (34.6)	255 (47.6)	
Total Family Income				<0.0001
< 25 k	111 (21.5)	83 (22.6)	166 (34.5)	
≥25 k and <50 k	167 (32.3)	105 (28.6)	143 (29.7)	
≥50 k and <75 k	87 (16.8)	73 (19.89)	94 (19.5)	
≥75 k	152 (29.4)	106 (28.9)	78 (16.22)	

	**General Perceived Stress Score**			**P for trend**^‡^

	**Low Stress** **(lowest tertile)**	**Medium Stress** **(middle tertile)**	**High Stress** **(highest tertile)**	

N	517	523	475	

Age				0.01
< 50 years	171 (34.1)	204 (39.5)	203 (43.2)	
≥50 years	331 (65.9)	312 (60.5)	267 (56.8)	
Gender				0.8
Men	162 (31.3)	150 (28.7)	115 (24.2)	
Women	355 (68.7)	373 (71.3)	360 (75.8)	
BMI, kg/m^2^				0.06
≤25	130 (27.0)	127 (25.8)	103 (22.5)	
>25 and <30	186 (38.6)	162 (32.9)	160 (34.9)	
≥30	166 (34.4)	203 (41.3)	195 (42.6)	
Smoking Status				0.004
Current Smoker	64 (12.5)	61 (11.7)	105 (22.2)	
Former Smoker	120 (23.4)	110 (21.1)	74 (15.6)	
Non-smoker	329 (64.1)	350 (67.2)	295 (62.2)	
Physical Activity^§^				0.003
Yes	407 (79.2)	398 (76.2)	316 (66.7)	
No	107 (20.8)	124 (23.8)	158 (33.3)	
Education^#^				<0.0001
Less than HS	34 (6.8)	40 (7.8)	67 (14.3)	
HS graduate	90 (19.9)	112 (21.8)	158 (33.8)	
More than HS	377 (15.3)	362 (70.4)	243 (51.9)	
Hypertension^†^				0.2
Yes	274 (53.1)	292 (56.0)	293 (61.9)	
No	242 (46.9)	229 (44.0)	180 (38.1)	
Diabetes^†^				0.7
Yes	78 (15.1)	82 (15.7)	94 (20.0)	
No	438 (84.9)	439 (84.3)	377 (80.0)	
High Cholesterol^†^				0.7
Yes	190 (37.1)	196 (37.7)	194 (41.5)	
No	322 (62.9)	324 (62.3)	273 (58.5)	
History of MI^†^				0.03
Yes	27 (5.2)	34 (6.5)	34 (7.2)	
No	488 (94.8)	489 (93.5)	438 (92.8)	
Financial Stress				<0.0001
Lowest tertile	281 (55.9)	170 (32.9)	65 (13.8)	
Middle tertile	154 (30.7)	194 (37.6)	118 (25.1)	
Upper tertile	67 (13.4)	152 (29.5)	287 (61.1)	
Total Family Income				<0.0001
< 25 k	75 (16.3)	118 (25.4)	163 (38.4)	
≥ 25 k and < 50 k	138 (30.0)	127 (27.3)	143 (33.6)	
≥ 50 k and < 75 k	98 (21.3)	90 (19.3)	63 (14.8)	
≥ 75 k	149 (32.4)	130 (28.0)	56 (13.2)	

	**Sleep Duration Categories**^**±**^			**P for trend**^‡^

	**> 7 hrs**	**6-7 hrs**	**5-6 hrs**	**< 5 hrs**

N	307	341	707	157

Age				0.003
< 50 years	115 (37.5)	129 (37.8)	279 (39.5)	56 (35.7)
≥ 50 years	192 (62.5)	212 (62.2)	428 (60.5)	101 (64.3)
Gender				0.2
Men	74 (24.1)	106 (31.1)	207 (29.3)	39 (24.8)
Women	233 (75.9)	235 (68.9)	500 (70.7)	118 (75.2)
BMI, kg/m^2^				0.03
≤ 25	90 (30.6)	88 (27.0)	165 (24.2)	30 (19.7)
> 25 and < 30	104 (35.4)	122 (37.4)	240 (35.2)	47 (30.9)
≥ 30	100 (34.0)	116 (35.6)	277 (40.6)	75 (49.4)
Smoking Status				0.04
Current Smoker	52 (17.0)	42 (12.4)	102 (14.5)	33 (21.2)
Former Smoker	53 (17.3)	82 (24.2)	147 (20.9)	22 (14.1)
Non-smoker	201 (65.7)	215 (63.4)	455 (64.6)	101 (64.7)
Physical Activity^§^				0.03
Yes	226 (74.1)	266 (78.0)	523 (74.3)	103 (65.6)
No	79 (25.9)	75 (22.0)	181 (25.7)	54 (34.4)
Education^#^				0.07
Less than HS	35 (11.4)	32 (9.4)	57 (8.1)	25 (16.0)
HS graduate	77 (25.2)	81 (23.7)	170 (24.1)	41 (26.3)
More than HS	194 (63.4)	228 (66.9)	477 (67.8)	90 (57.7)
Hypertension^†^				0.3
Yes	166 (54.3)	191 (56.0)	399 (56.8)	100 (63.7)
No	140 (45.7)	150 (44.0)	304 (43.2)	57 (36.3)
Diabetes^†^				0.004
Yes	58 (19.1)	46 (13.5)	109 (15.5)	40 (25.5)
No	246 (80.9)	295 (86.5)	594 (84.5)	117 (74.5)
High Cholesterol^†^				0.06
Yes	108 (35.3)	117 (34.5)	286 (41.0)	68 (44.2)
No	198 (64.7)	222 (65.5)	411 (59.0)	86 (55.8)
History of MI^†^				0.007
Yes	24 (7.9)	10 (2.9)	44 (6.2)	16 (10.3)
No	281 (92.1)	331 (97.1)	662 (93.8)	139 (89.7)
Financial Stress				<0.0001
Lowest tertile	118 (38.6)	141 (41.4)	237 (33.5)	33 (21.0)
Middle tertile	102 (33.3)	113 (33.1)	214 (30.3)	42 (26.8)
Upper tertile	86 (28.1)	87 (25.5)	256 (36.2)	82 (52.2)
Total Family Income				<0.0001
< 25 k	85 (31.4)	67 (21.8)	151 (23.3)	55 (40.7)
≥ 25 k and < 50 k	86 (34.0)	86 (28.0)	201 (31.0)	36 (26.7)
≥ 50 k and < 75 k	37 (13.6)	64 (20.9)	130 (20.0)	23 (17.0)
≥ 75 k	57 (21.0)	90 (29.3)	167 (25.7)	21 (15.6)

* Number and percentage for categorical variables; mean and standard deviation for continuous variables;

^§^Self reported physical activities or exercises such as running, calisthenics, golf, gardening or walking (outside of regular job);

^†^Ever told by a doctor, nurse or any health care provider;

^‡^Calculated with a chi-square test;

^#^Classified in less than a high school (HS) graduate, a HS graduate and more than a HS graduate;

^±^Three participants did not answer the related question.

GSQ continuous score was modestly but significantly associated with BMI continuous, (a correlation coefficient, r = 0.13; indicating a worse global sleep quality associated with a higher BMI), and moderately and significantly with GPS, r = 0.36, indicating a higher global perceived stress associated with a worse sleep quality (Table 2). Sleep duration (continuous) and use of sleeping medications showed a modest association with BMI (Table 2). Sleep duration, sleep latency and use of sleeping medication showed a moderate association with GPS (Table 2). BMI had a moderate association with sleep disturbances (r = 0.33, p < 0.0001) and daytime dysfunction (r = 0.35, p < 0.0001). The partial correlations (with adjustment for age and gender) between GSQ and BMI was 0.19 (p < 0.0001), and between GPS and BMI was 0.09 (p = 0.001).

**Table 2:** Correlation coefficients between the main variables

	Global Sleep Quality	Sleep Duration (contin.)	Sleep Latency	Sleeping Medication	General Perceived Stress	Body Mass Index
Global Sleep Quality	******	-0.65* <.0001^§^	0.70 <.0001	0.45 <.0001	0.36 <.0001	0.13 <.0001
Sleep Duration (continuous^†^)		******	-0.27 <.0001	-0.09 0.0006	-0.16 <.0001	-0.12 <.0001
Sleep Latency			******	0.29 <.0001	0.26 <.0001	0.04 0.17
Use of Sleeping Medication				******	0.18 <.0001	0.08 0.004
General Perceived Stress					******	0.08 0.002

*Spearman correlation coefficient;

^§^P-value for statistical significance;

^†^Considered continuously.

The multivariable-adjusted odds ratios of obesity for the sleep quality components are presented in Table 3. Gender was an effect modifier of the association between habitual sleep efficiency (p = 0.02) and daytime dysfunction (p = 0.04) with obesity (Table 3). Among women, after adjustment for age, physical activity, smoking status, education and history of hypertension, hypercholesterolemia, diabetes and myocardial infarction, there was an increased likelihood of obesity for those participants with a higher (worse) sleep disturbance score [OR (95% C.I.): 1.56 (1.24 - 1.97)] and with a higher (worse) daytime dysfunction score [OR (95% C.I.): 1.39 (1.10 - 1.76)]. For sleep disturbance, the odds ratios remained significant after further adjustment for general perceived stress [OR (95% C.I.): 1.55 (1.22 - 1.98)] or for total family income, [OR (95% C.I.): 1.48 (1.16 - 1.89)] (Table 3). For daytime dysfunction, the odds ratios remained significant after further adjustment for GPS, but not after further adjustment for total family income (Table 3).

**Table 3:** Multivariable-adjusted odds ratios of obesity for sleep quality domains by gender

		Women		Men		
		**OR**	**95% CI**	**OR**	**95% CI**	**p-value for gender interaction**

Sleep Quality	MV-adjusted*	1.12	0.93 - 1.36	0.78	0.50 - 1.20	0.09
	GPS-adjusted^§^	1.06	0.87 - 1.30	0.73	0.45 - 1.17	0.08
	FS-adjusted^†^	1.04	0.85 - 1.27	0.75	0.47 - 1.21	0.08
Sleep Latency	MV-adjusted	1.12	0.95 - 1.31	0.99	0.75 - 1.30	0.38
	GPS-adjusted^§^	1.08	0.92 - 1.27	0.96	0.73 - 1.26	0.30
	FS-adjusted^†^	1.06	0.90 - 1.25	0.96	0.73 - 1.27	0.30
Sleep Duration	MV-adjusted	1.20	1.00 - 1.43	0.90	0.63 - 1.27	0.10
	GPS-adjusted^§^	1.16	0.97 - 1.39	0.86	0.60 - 1.22	0.07
	FS-adjusted^†^	1.14	0.95 - 1.37	0.88	0.61 - 1.27	0.07
Habitual Sleep Efficiency	MV-adjusted	1.15	0.99 - 1.33	0.81	0.59 - 1.11	0.02
	GPS-adjusted^§^	1.13	0.98 - 1.31	0.78	0.56 - 1.07	0.02
	FS-adjusted^†^	1.12	0.97 - 1.29	0.78	0.56 - 1.08	0.02
Sleep Disturbance	MV-adjusted	1.56	1.24 - 1.97	1.31	0.86 - 1.99	0.20
	GPS-adjusted^§^	1.55	1.22 - 1.98	1.30	0.82 - 2.07	0.19
	FS-adjusted^†^	1.48	1.16 - 1.89	1.33	0.84 - 2.10	0.19
Use of Sleeping Medication	MV-adjusted	1.16	0.97 - 1.37	1.30	0.91 - 1.85	0.85
	GPS-adjusted^§^	1.13	0.95 - 1.35	1.32	0.90 - 1.94	0.86
	FS-adjusted^†^	1.11	0.94 - 1.32	1.32	0.90 - 1.95	0.86
Daytime Dysfunction	MV-adjusted	1.39	1.10 - 1.76	0.82	0.52 - 1.31	0.04
	GPS-adjusted^§^	1.36	1.05 - 1.77	0.79	0.48 - 1.30	0.04
	FS-adjusted^†^	1.30	1.00 - 1.68	0.82	0.51 - 1.32	0.04

*With adjustment for age, physical activity, smoking status, education and history of hypertension, hypercholesterolemia, diabetes and myocardial infarction (multivariable-adjusted);

^§ ^Multivariable-adjusted (MV-adjusted) with additional for GPS (general perceived stress);

^† ^MV-adjusted with additional for FS (financial stress).

After multivariable adjustment that included age, gender, physical activity, smoking status, education, total family income, financial stress and history of hypertension, hypercholesterolemia, diabetes and myocardial infarction, sleep quality as assessed by GSQ continuous score showed an association with obesity among women [OR (95% C.I.): 1.08 (1.03 - 1.12)] but not among men [OR (95% C.I.): 0.98 (0.89 - 1.09)]. Continuous BMI showed an association with GPS (p = 0.02) when categorized into tertiles.

In crude models, GSQ categorized score did not interact with GPS (categorized into tertiles) to significantly increase the likelihood of obesity (p = 0.85). However, GPS categorized positively interacted with GSQ continuous to significantly increase the likelihood of obesity (p = 0.02). Our data suggest that there was an increased likelihood of obesity in the medium stressed participants (odds ratio of 1.09 [1.02 - 1.17]) compared to the other two groups (1.01 [0.94 - 1.10], for low stress, and 1.03 [0.97 - 1.10], for high stress), and therefore stress appears to modulate the association between obesity and sleep quality.

After adjustment for age, gender, physical activity, smoking status, education, financial stress, total family income and history of hypertension, hypercholesterolemia, diabetes and myocardial infarction, no significant interactions were detected between stress and the PSQI components on obesity.

## Discussion

In our cross-sectional investigation we found that global sleep quality and sleep duration were associated with categorized body mass index (normal weight, overweight and obesity). In women but not in men, after multivariable adjustment, sleep quality as assessed by the global sleep quality continuous score showed an association with obesity. Among women, sleep disturbance and daytime dysfunction, two of the PSQI components, were also significantly associated with obesity in multivariable-adjusted models. The association of continuous sleep quality with obesity was modified by perceived stress categorized into tertiles. There appears to be an increased likelihood of obesity in the medium stress group of participants compared with the other two groups.

Sleep disorders are prevalent and yet underexplored [39]. According to the National Sleep Foundation survey, 39% of American adults obtain less than the recommended 7 hours of sleep per weeknight [40]. Obstructive sleep apnea, for example, is a very common disease whose population prevalence is comparable to that of other chronic diseases such as asthma, chronic obstructive pulmonary disease, type 2 diabetes and coronary heart disease [41]. Sleep disturbance appears to be more prevalent in African Americans than whites [15,16,39,42]. Therefore, our finding that half of the participants reported sleeping less than six hours per night was an anticipated result. Noteworthy was the association of gender, physical activity, smoking, education, financial stress, family income and history of myocardial infarction with sleep quality, in concordance with previous studies [43,44]. As gender has been previously associated with sleep disturbance[31-33], the emergence of gender as an effect modifier for the association of obesity with GSQ and PSQI components was not surprising.

Research suggests chronic sleep restriction impairs cognitive function as well as influence cardiovascular and metabolic health [30]. Sleep deprivation has several adverse physiological consequences, including impaired glucose tolerance and insulin sensitivity, elevated sympathetic tone, increased inflammation, and the increase of ghrelin and the decrease of leptin with the subsequent increase of hunger and appetite [45-50]. There is a growing body of literature that places sleep disorders upstream on the causal pathway of obesity [19,51-53]. In multivariate analyses including adjustment for BMI, both the Nurses Health Study as well as the NHANES identified short sleep as a risk factor for incident diabetes [54,55]. As obesity is distributed differentially by ethnic groups and given its burden is growing, an increase of sleep disorders may further deepen the ethnic disparity in obesity-related cardiovascular disease (CVD). The comparison of our study with studies such as NHANES I[19], Zurich cohort[20], CARDIA[16] and Nurses Health Study[21] that reported an association between sleep duration and obesity is challenging as those cohorts' participants have a lower average BMI.

Psychosocial stress, which has been shown to affect cardiovascular endpoints differently in ethnic minorities [56-59], was also shown to be inversely associated with quality of sleep [27,28]. When sleep quality global score was considered continuous, we found an interaction between sleep quality and stress. There appears to be an increased risk of obesity in the medium stress group compared to the other two groups. To our knowledge, the other few studies that assessed both sleep quality and psychological stress[60-62] did not consider their interaction. Our data suggest that stress may modulate the association between obesity and sleep quality. Total family income and financial stress were also associated with sleep duration in accordance with previous studies [63]. Moreover, general perceived stress and financial stress diminished the significant associations of sleep disturbance and daytime dysfunction with obesity to the point of non-significance, suggesting that stress mediates the association between these PSQI components and obesity. Therefore, simultaneous assessment of general stress and financial stress should be attempted when investigating the association of sleep components with obesity.

Questionnaires such as Berlin questionnaire[64,65] or Pittsburgh questionnaire remain the instruments of choice for telephone surveys and epidemiological studies without clinical exams, for which a more objective sleep assessment using polysomnography or actigraphy devices would be desirable. Our finding that only two of the PSQI domains, sleep disturbance and daytime dysfunction, were associated with obesity in multivariable-adjusted models indicate the need for a full query of sleep quality domains.

The current study is among the first to assess in AA the interaction of sleep quality with psychosocial stress on obesity with adjustment for physical activity, a major correlate of obesity, albeit a limited physical activity assessment (which constitutes one of the main limitation of our investigation). Similarly with other telephone surveys, the majority of respondents were female which limits the generalizability to AA males. Other notable limitations are the facts that BMI and sleep assessment are only self-reported measures and there was no dietary intake information or depression symptoms collected. Among the strengths of our investigation are a thorough assessment of psychosocial and financial stress and a complete Pittsburgh sleep questionnaire.

Epidemiological research shows that short self-reported sleep duration is associated with several endpoints such as diabetes, coronary heart disease and mortality [18,30]. Part of these associations might be mediated by obesity. Existing data have variable consistency. Twenty years after the development of the Pittsburgh Sleep Quality Index, few other questionnaires have been specifically designed to measure sleep quality. The PSQI remains a sensitive tool when queried entirely and when used in conjunction with assessments of different types of stress.

## Conclusions

The association of sleep quality with obesity was modified by perceived stress; among those with medium stress, there was an association between sleep quality and obesity. Simultaneous assessments of sleep and stress should be performed whenever possible.

## Competing interests

The authors declare that they have no competing interests.

## Authors' contributions

AB, RD-D and VLW conceived of and designed the study. AB and ZC performed the statistical analyses. AB, RD-D, DLC, SGB, GHG and VLW interpreted the results. AB, RD-D and VLW drafted the manuscript. All authors revised the manuscript for intellectual content, and read and approved the final manuscript.

## Pre-publication history

The pre-publication history for this paper can be accessed here:

http://www.biomedcentral.com/1471-2458/10/581/prepub
